# The relationship between motor competence and executive function as influenced by age, sex, and family socio-economic status

**DOI:** 10.3389/fpsyg.2025.1544168

**Published:** 2025-02-27

**Authors:** Behrouz Ghorbanzadeh, Behzad Mohammadi Orangi, Tolga Sahin

**Affiliations:** ^1^Department of Physical Education and Sport Sciences, Faculty of Education and Psychology, Azarbaijan Shahid Madani University, Tabriz, Iran; ^2^Department of Sport Science, School of Humanities, Damghan University, Damghan, Iran; ^3^Dokuz Eylul University, Necat Hepkon Sports Sciences, Izmir, Türkiye

**Keywords:** motor competence, executive function, socio-economic status, age, sex

## Abstract

**Introduction:**

Motor Competence (MC) plays a fundamental role in physical, cognitive, and social development, while executive function (EF) is a key factor influencing MC. The primary objective of this study was to compare MC across three age groups (children, adolescents, and young adults), sex, and socio-economic status (SES). The main aim was to investigate the relationship between MC and EF and to determine whether age, sex, and SES could modify this relationship.

**Methods:**

This descriptive-correlational study evaluated 475 participants from three age groups (8–11, 12–15, and 18–21 years). MC was measured using the BOT-2 test, and EF was assessed with the Stroop test. Data were analyzed using independent t-tests, one-way ANOVA, and linear regression analysis.

**Results:**

Findings revealed that MC was higher in children compared to adolescents and young adults, and higher SES was associated with better MC, whereas sex had no significant effect on MC. Additionally, a strong positive relationship (44%) was identified between EF and MC, with this relationship being moderated by age, sex, and SES.

**Discussion:**

The results indicated that MC and EF are influenced by the interaction of individual (age and sex) and environmental (SES) constraints. These findings underscore the importance of incorporating these factors into educational and sports planning for more holistic development.

## Highlights

- Children have higher motor competence (MC) compared to adolescents and young adults; higher socioeconomic status (SES) improves MC, while gender has no significant effect.
- There is a strong positive relationship (44%) between MC and executive function (EF), which is moderated by age, gender, and SES.
- Age, gender, and SES influence the relationship between MC and EF and should be considered in educational and sports planning.

## 1 Introduction

Motor Competence (MC), as one of the critical dimensions of human development, plays a vital role in physical, social, and cognitive abilities (Medeiros et al., 2023). Individuals with high MC not only perform more successfully in sports activities but also experience higher self-confidence (Medeiros et al., 2023), better academic performance (Ghorbanzadeh and Aghdasi, 2020), and greater social growth and participation (Capio et al., 2024). Longitudinal studies suggest that MC is not just associated with academic achievement, but may also serve as a mediator in cognitive development. Specifically, studies have shown that motor skills, particularly fine and gross motor skills, contribute to the development of cognitive abilities such as working memory, attention, and executive function, which in turn positively influence academic performance (Macdonald et al., 2018; Zhou and Tolmie, 2024). For example, fine motor proficiency has been linked to better academic outcomes in mathematics and reading, while gross motor skills like speed, agility, and upper-limb coordination have been shown to enhance cognitive function (Macdonald et al., 2018; Zhou and Tolmie, 2024). These associations are believed to stem from the fact that motor competence influences neural development, which is crucial for cognitive and academic performance. Conversely, weaknesses in these skills can lead to reduced physical activity, a decline in mental health, and an increased risk of diseases such as obesity (De Meester et al., 2016). Therefore, identifying and strengthening the factors influencing MC is of particular importance, as these factors can improve both cognitive and physical health, thereby enhancing individuals’ overall quality of life (Cattuzzo et al., 2016).

Individual, environmental, and task constraints according to the ecological dynamics perspective (Button et al., 2020), influence motor development, and consequently MC. This perspective emphasizes the interaction of these constraints in shaping motor abilities. In other words, based on this view, motor development and the enhancement of MC arise from the interaction of individual constraints such as age, sex, or EF; environmental constraints such as the family’s socio-economic status (SES); and task constraints such as the rules of each skill (Button et al., 2020).

Previous studies have highlighted the role of individual constraints on motor coordination (MC). Specifically, Mohammadi Orangi et al. (2017, 2018, 2023a,b) have explored how factors such as age, body mass index (BMI), and nutritional status influence MC. For instance, the relationship between MC and IQ has been shown to vary with age, being stronger in childhood and adolescence but weaker in adulthood. Similarly, the effect of BMI on MC changes across different age groups, and the influence of nutritional status on MC evolves from childhood to adulthood. These findings collectively offer valuable insights into the dynamic nature of individual constraints on MC.

However, in these studies, all participants were male, leaving the role of sex unexplored despite its potential influence. Supporting this idea, Jiménez-Díaz et al. (2015) found that age and sex, as individual constraints, significantly impact motor skills. Recently, Mohammadi Orangi et al. (2023a,b) demonstrated that individuals with higher emotional intelligence display better MC. The study also identified a positive correlation between MC and emotional intelligence, although this relationship was influenced by both age and sex. While age and sex were considered as individual constraints affecting the relationship between MC and emotional intelligence, including an environmental constraint alongside these factors could offer researchers and coaches a more comprehensive perspective on coaching, talent identification, and the analysis of factors related to MC. Furthermore, the studies mentioned selected participants purposefully, such as 30 children with high emotional intelligence, 30 with low emotional intelligence, and 30 with average emotional intelligence. This approach limits the generalizability of the findings to diverse populations (Mohammadi Orangi et al., 2023a,b). Therefore, replicating these studies while considering a broader range of constraints would be beneficial.

One of the individual constraints affecting motor development is executive function (EF) (Koziol and Lutz, 2013). EF encompasses a set of cognitive processes essential for guiding goal-directed behaviors (Blair, 2017). Since motor behavior and the EF of the brain are interconnected, the influence of EF on motor behavior is evident. EF helps individuals prepare for movement execution and make different decisions regarding movement in various situations (McClelland and Cameron, 2019). Therefore, the relationship between EF and MC can be explained. A review of the literature also demonstrates that EF is associated with MC (Albuquerque et al., 2022). In a study conducted on rural children in Iran, results indicated a significant relationship between EF and gross motor skills (Fathirezaie et al., 2022). Additionally, a large meta-analysis of 32 studies revealed that balance and manual skills, as components of MC, have a significant relationship with EF (Fathirezaie et al., 2022). These findings highlight that EF is another constraint that can be associated with MC.

Although the relationship between MC and EF has been confirmed in some studies (Fathirezaie et al., 2022), it has not been comprehensively examined across different age and sex groups (children, adolescents, and young adults). In addition to age and sex, which are individual constraints, SES is an environmental constraint that can influence the relationship between EF and MC (Chang and Gu, 2018). SES typically considers parental education, occupation, and income levels (Cook et al., 2019). Research has shown that low parental SES is associated with lower MC in children, adolescents, and young adults (ages 4–17) (Möller et al., 2021). Therefore, investigating its impact on the relationship between MC and EF, alongside individual constraints like age and sex, can enhance current knowledge in motor development, given the limitations of existing studies. This is especially important because SES has been shown to affect both EF (Vrantsidis et al., 2020) and MC (Morley et al., 2015).

Based on the discussed topics, the primary aim of this study is to examine the influence of individual (age, sex) and environmental (socioeconomic status or SES) factors on motor competence (MC). This study specifically investigates the relationship between MC and executive function (EF), and whether the potential effects of age, sex, and SES on MC also modify the relationship between MC and EF. While numerous studies have explored the impact of individual and environmental factors on MC and EF, significant gaps remain in the existing literature. In particular, most prior research has focused on specific groups of participants, such as those with high or low emotional intelligence, limiting the generalizability of the findings to broader populations. Furthermore, the influence of SES on the relationship between MC and EF has not been comprehensively examined.

This study adopts the ecological dynamics perspective and constraints theory to guide its hypotheses. According to ecological dynamics (Button et al., 2020), motor competence (MC) is shaped by the interaction of individual, environmental, and task constraints. This framework emphasizes that motor development arises from the continuous interaction of these constraints, rather than isolated factors, influencing an individual’s ability to perform motor tasks. Specifically, we hypothesize that individual constraints, such as age and sex, will directly affect MC, in line with ecological dynamics theory, and task and environmental constraints, like SES, will further modulate MC. Additionally, the relationship between MC and EF may be influenced by these constraints, with the hypothesis suggesting that the interaction between age, sex, and SES affects how EF contributes to motor performance. By incorporating these factors, this study aims to fill existing gaps, providing a more comprehensive understanding of how these variables interact and affect both motor competence and executive function. This approach can advance knowledge in motor development and offer practical insights for coaches, talent analysts, and policymakers.

## 2 Methodology

This study employed a descriptive and correlational design to examine the relationship between MC and executive function (EF), considering the effects of age, sex, and socio-economic status (SES) on motor competence (MC).

### 2.1 Participants

The population for this study included children aged 8–11 years, adolescents aged 12–15 years, and young adults aged 18–21 years. The minimum sample size was estimated using the G*Power 3.1.9.4 software (F tests—Linear multiple regression: Fixed model, *R*^2^ deviation from zero). Two variables were considered as criterion variables: MC and EF. Additionally, the effect size was estimated at 0.15, with an alpha error probability of 0.05 and a test power of 0.95. In this calculation, an effect size of 0.15 was selected based on a review of existing literature (Fathirezaie et al., 2022). This effect size is commonly used in similar studies to analyze relationships between MC and EF and is considered a small to moderate effect size. Thus, the selected effect size provides an adequate statistical power to detect meaningful differences (Fathirezaie et al., 2022). Based on these parameters, the minimum required sample size for this study was 107 participants. Accordingly, at least 107 participants were needed for each age group. However, considering potential dropout and participant withdrawal, which are common in research studies (Ghanati et al., 2022; Letafatkar et al., 2020), we decided to recruit a larger sample size.

**Figure 1:**
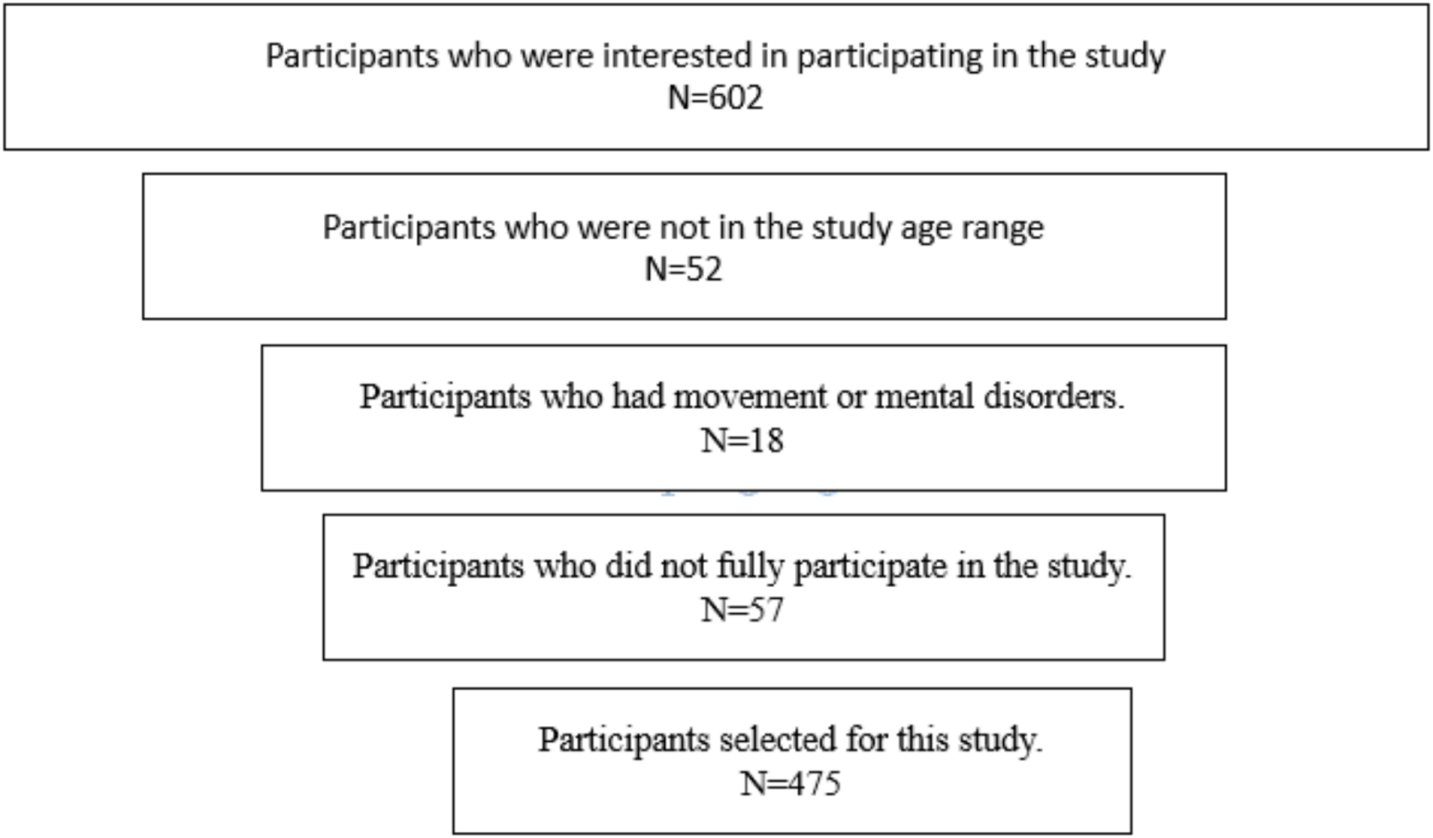
How participants are selected.

To select participants, an announcement was published in the city of Izmir, Turkey, outlining the study’s objectives and inclusion and exclusion criteria. This announcement was also shared in virtual groups. Based on this information, interested participants contacted the authors and visited a predetermined location at University for evaluation. Initially, 602 individuals expressed interest in participating in the study across three age groups. However, 127 of these individuals did not meet the necessary criteria, leaving 475 participants who were evaluated. A general diagram illustrating the sample selection process can be found in Figure 1. The inclusion criteria for the study were as follows: 1. being aged 7–11 for children, 12–15 for adolescents, and 18–21 for young adults; 2. having full physical and mental health verified by health records (for school and university students); and 3. participating fully in all designated evaluations. The criterion of “full physical and mental health” refers to the verification of participants’ health status through their health records. For children and adolescents, this criterion was assessed based on school health records, which include details such as general physical health, history of chronic illnesses, previous injuries, and any medical conditions that could potentially impact participation in physical activities or cognitive tasks. For young adults, this information was confirmed through university health records, including similar details on physical health and any significant medical history. Only individuals with no reported conditions that would interfere with their physical or cognitive performance were included in the study. If any health issues were identified during the initial evaluation, participants were excluded from the study. The exclusion criterion was incomplete participation in any of the evaluations. Moreover, the selection of age groups was based on previous research (Mohammadi Orangi et al., 2023a,b) in the field of motor and cognitive development. Studies have shown that middle childhood (ages 8–11) is a period when fundamental motor skills become consolidated, whereas adolescence (ages 12–15) is considered a critical stage for the development of executive function due to hormonal changes and cognitive growth. On the other hand, young adulthood (ages 18–21) provides an opportunity to examine the stability of these skills in later stages of life. This selection allows researchers to analyze developmental changes across different time spans and better assess the potential influence of environmental and individual factors (Mohammadi Orangi et al., 2023a,b).

Before commencing the study, written consent was obtained from all participants aged 18 and older, as well as from the parents of participants under the age of 18.

### 2.2 Measuring tools

#### 2.2.1 EF: Stroop test

EF was measured using the Stroop test, a test first created by Stroop (1935) and considered one of the earliest and most effective tests for studying EF. Many studies have utilized this test to assess EF(Braga et al., 2022; Faria et al., 2015; Phillips et al., 2002), and it is globally recognized for its high validity and reliability (Savaş et al., 2020). In this research, a computerized version of the test was used. During the Stroop test, participants were shown 48 congruent and 48 incongruent color words in red, blue, yellow, and green. Congruent words matched the color and meaning (e.g., “green” in green), while incongruent words had a mismatch (e.g., “green” in red, blue, or yellow). A total of 96 congruent and incongruent color words were randomly and sequentially displayed. Participants were instructed to respond based solely on the color shown, ignoring the word’s meaning, and were made aware that the words may not match their colors. The task was to identify only the color of the words. Each stimulus appeared on the screen for 2 s with an 800-millisecond interval between stimuli. Accuracy, based on the number of correct answers, was used to score the test. The retest reliability of this test is 0.60 and 0.97, respectively.

#### 2.2.2 MC, Broninx-Ozertsky motor skills test BOT-2 (short form)

The BOT-2 Bruininks-Oseretsky Test of MC Ed. 2 (short form) was utilized to assess MC. This test comprises 8 sub-tests, with 4 sub-tests focusing on gross movements, 3 on fine movements, and 1 on upper body coordination, totaling 46 items that cover a wide range of high-quality movement skills. The test offers a comprehensive measure of MC, along with individual scales for fine and gross motor skills for individuals aged 4–21. The long form of the test takes 45–60 min to complete, while the short form can be completed in 15–20 min, making it a useful screening tool. The test has demonstrated validity and reliability, with a reliability coefficient of 90% for motor skills assessment. The retest reliability coefficients are 0.78 for the long form and 0.86 for the short form. The short form assesses overall motor skills, with the total score reflecting a combination of gross and fine skills (Bruininks, 1978). Previous studies have also used this test to evaluate motor skills (Kose et al., 2021). In this study, standard scores were utilized, with the total score serving as the criterion (Ghorbanzadeh et al., 2024; Mohammadi Orangi et al., 2023a,b).

The BOT-2 test results were scored using standard scores, which are typically derived from the raw scores obtained on each of the subtests. These raw scores were converted into standard scores that reflect the participant’s performance relative to the normative sample. Specifically, the total standard score was used as the criterion variable in the analysis, which combines scores from both gross and fine motor skills, as well as upper body coordination. The standard scores allow for a more meaningful comparison of an individual’s motor competence (MC) to a broader population of their same age group, making them appropriate for assessing group differences and individual abilities (Ghorbanzadeh et al., 2024; Mohammadi Orangi et al., 2023a,b).

#### 2.2.3 SES

Participants’ SES was assessed using a combination of parental education (graduate and professional education), employment status, and household equalized monthly income. The final SES score could range from 3 to 21, with higher scores indicating higher SES. SES scores could be categorized as low, moderate, or high (Möller et al., 2021; Morley et al., 2015).

### 2.3 Research implementation method

To conduct this study, participants were invited to attend a predetermined gymnasium from 8 am to 7 pm for 1 month at their convenience. Participants first completed a SES questionnaire, which included information such as age, sex, height, weight, and general educational and financial status of the family. Parents filled out the questionnaire for children and adolescents. Digital scales and height meters were used to accurately measure weight and height, with corrections made for any inconsistencies. Following this stage, participants were administered a Stroop test on a laptop in a room within the gym. Each person completed the test individually, with an expert familiar with the Stroop test overseeing the process. Specific instructions were provided to each participant before the test, with additional explanations given if needed. Next, the participants underwent the BOT-2 test, evaluated by two experts familiar with the assessment. A Sony Alpha a6700 body camera was used to film the test, allowing for comparison in case of significant score discrepancies between the evaluators. Only one person was allowed in the testing hall at a time. The standard scores from both the Stroop test and the BOT-2 test were provided to the authors for final evaluation and analysis. Finally, each participant received a sports T-shirt for their participation in the study.

### 2.4 Data management and analysis

Data was screened using the Shapiro–Wilk test and confirmed to be normally distributed (all *p* > 0.05). Independent t-tests were used to compare MC between boys and girls across childhood, adolescence, and young adulthood. One-way ANOVA tests examined differences in MC among the three SES groups and across age groups (children, adolescents, and young adults). The Bonferroni correction test was applied for multiple comparisons. Correlation analysis was conducted to assess the relationship between MC and EF (EF). Additionally, linear regression analysis was performed to evaluate the association between MC and EF in univariate analysis. All statistical analyses were conducted using SPSS 22 for Windows, with significance set at *p* < 0.05. Data is presented as mean ± SD.

## 3 Results

**Figure 2:**
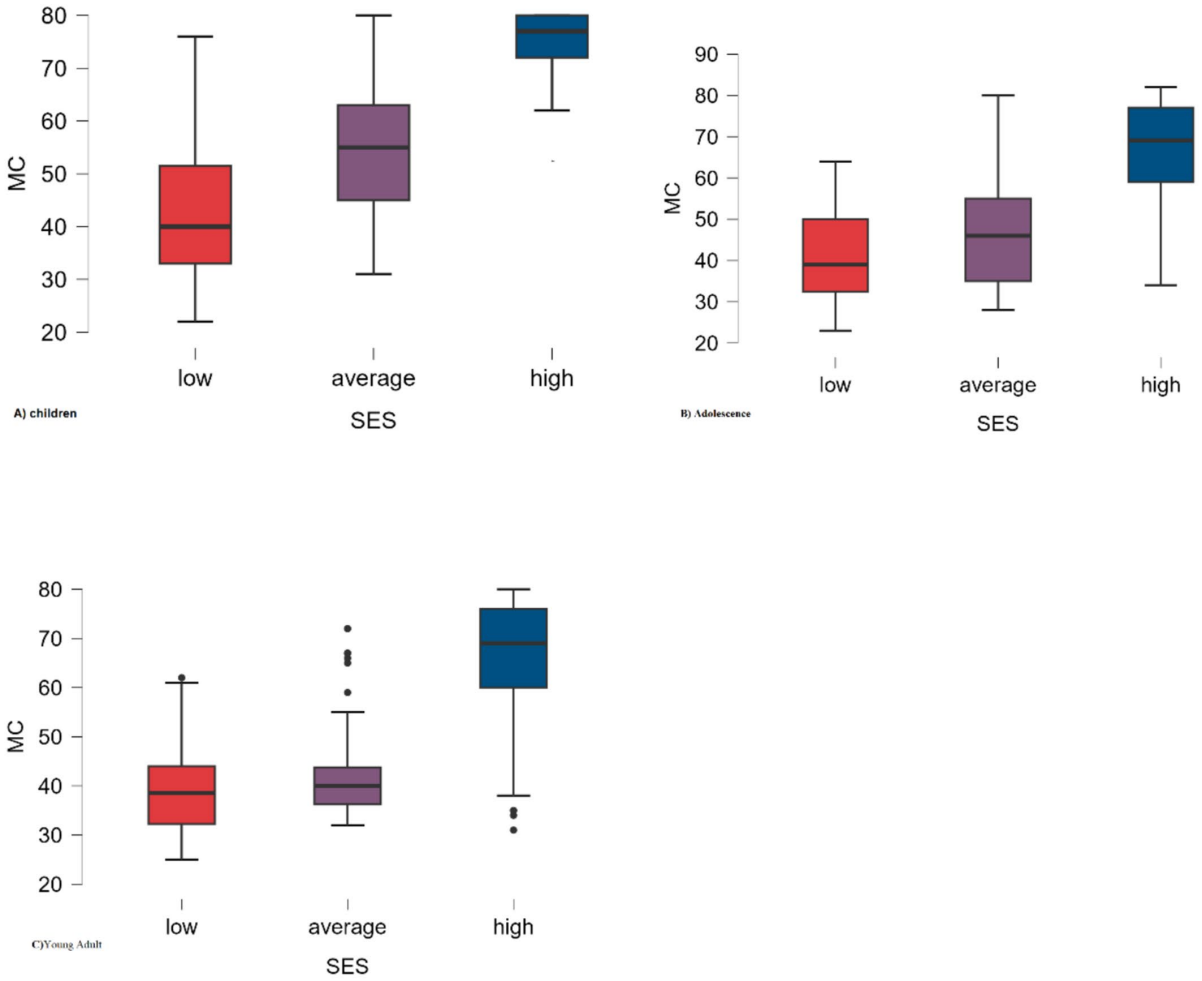
Participants’ MC based on their families’ SES.

Table 1 presents the demographic information of the participants, which included 154 children, 164 adolescents, and 157 young adults. This table also shows general information on motor competence (MC) and executive function (EF) in the three age groups, considering sex and Socio-Economic Status (SES). Figure 2, also, shows the motor skills MC of children, adolescents, and adults based on SES.

**Table 1:** Complete demographic information of participants.

			Age (Year)	Weight (kg)	High (cm)	MC	EF
			*N*, Mean ± SD	*N*, Mean ± SD	*N*, Mean ± SD		
Children	Girl	Low SES	24, 8.57 ± 0.93	24, 26.89 ± 1.64	24, 123.86 ± 3.55	44.00 ± 18.29	43.14 ± 16.68
		Moderate SES	27, 8.45 ± 0.78	27, 27.08 ± 1.54	27, 121.85 ± 2.61	54.56 ± 14.26	53.66 ± 21.01
		High SES	21, 8.25 ± 0.89	21, 27.43 ± 1.77	21, 121.14 ± 1.77	72.05 ± 7.24	72.56 ± 6.83
	Boy	Low SES	27, 8.44 ± 0.96	27, 24.51 ± 3.55	27, 121.21 ± 3.57	43.85 ± 10.86	42.35 ± 15.84
		Moderate SES	37, 8.38 ± 0.89	37, 22.10 ± 3.61	37, 118.93 ± 2.89	53.73 ± 10.00	53.45 ± 14.70
		High SES	18, 8.57 ± 0.78	18, 20.47 ± 2.25	18, 118.09 ± 2.03	77.83 ± 3.73	74.16 ± 5.89
Adolescence	Girl	Low SES	21, 13.88 ± 0.81	21, 48.29 ± 2.77	21, 152.88 ± 3.98	47.48 ± 12.83	53.77 ± 16.99
		Moderate SES	40, 13.76 ± 0.89	40, 47.08 ± 2.76	40, 153.49 ± 4.95	42.43 ± 12.02	50.43 ± 17.19
		High SES	26, 13.32 ± 1.01	26, 46.36 ± 3.33	26, 153.42 ± 4.96	63.46 ± 15.41	67.03 ± 15.04
	Boy	Low SES	22, 13.30 ± 0.79	22, 63.23 ± 13.65	22, 162.48 ± 11.03	35.59 ± 9.19	37.18 ± 10.84
		Moderate SES	31, 13.20 ± 0.93	31, 52.39 ± 11.95	31, 152.72 ± 10.33	51.68 ± 10.65	56.84 ± 16.19
		High SES	24, 13.35 ± 1.01	24, 51.41 ± 12.18	24, 150.15 ± 10.79	68.88 ± 10.13	66.95 ± 10.48
Young adult	Girl	Low SES	25, 19.64 ± 1.02	25, 54.14 ± 3.11	25, 159.86 ± 3.22	40.56 ± 8.86	49.53 ± 17.19
		Moderate SES	28, 19.42 ± 0.70	28, 53.91 ± 3.48	28, 160.25 ± 2.64	42.89 ± 10.51	49.53 ± 17.19
		High SES	17, 19.60 ± 0.79	17, 53.87 ± 3.52	17, 160.18 ± 3.41	61.24 ± 16.60	63.25 ± 19.54
	Boy	Low SES	33, 19.40 ± 0.99	33, 81.89 ± 10.88	33, 182.51 ± 6.02	38.88 ± 9.47	39.47 ± 13.76
		Moderate SES	30, 19.27 ± 0.86	30, 80.96 ± 9.37	30, 183.17 ± 7.74	43.33 ± 10.80	50.52 ± 19.53
		High SES	24, 19.59 ± 0.99	24, 80.87 ± 10.38	24, 181.83 ± 7.31	68.50 ± 11.22	69.68 ± 11.95

According to Table 2, independent t-tests indicate that there is no significant difference between girls and boys in MC during childhood (*t* = 0.137, *p* = 0.891), adolescence (*t* = −0.993, *p* = 0.322), and young adulthood (*t* = −0.837, *p* = 0.404). Additionally, based on the one-way ANOVA test, a significant difference was found between the three SES groups (high>moderate>low) in children (*F* = 75.68, *p* = 0.001, η^2^ = 0.501), adolescents (*F* = 52.33, *p* = 0.001, η^2^ = 0.394), and young adults (*F* = 72.87, *p* = 0.001, η^2^ = 0.486). Regardless of age and sex, a significant difference was observed in the MC of the three groups (children>adolescents>young adults) across all age groups (*F* = 10.38, *p* = 0.001, η^2^ = 0.042).

**Table 2:** Results of independent *t*-test for comparing MC of girls and boys and one-way ANOVA test for comparing MC in three SES groups and age groups.

				Mean difference (I-J)	Std. error	Sig.	95% Confidence interval		Effect size
							Lower bound	Upper bound	Cohen’s *d*
Sex	Children		Boy*Girl	0.371	2.70	0.891	−4.97	5.71	0.022
	Adolescence		Boy*Girl	−2.51	2.52	0.322	−7.50	2.48	−0.155
	Young adult		Boy*Girl	−2.07	2.47	0.404	−6.96	2.81	−0.134
SES	Children	High	Low	30.79	2.52	0.001	24.82	36.77	−2.592
			Moderate	20.64	2.41	0.001	14.93	26.35	−1.730
		Low	Moderate	−10.15	2.22	0.001	−15.43	−4.88	−0.855
	Adolescence	High	Low	24.66	2.63	0.001	18.44	30.89	−1.949
			Moderate	19.59	2.33	0.001	14.07	25.12	−1.548
		Low	Moderate	−5.06	2.44	0.099	−10.85	0.072	−0.401
	Young adult	High	Low	25.88	2.26	0.001	20.52	31.25	−2.330
			Moderate	22.36	2.26	0.001	17.00	27.73	−2.013
		Low	Moderate	−3.51	2.06	0.207	−8.40	1.37	0.271
Age	Children	Adolescence		4.82	1.80	0.021	0.59	9.08	−5.561
		Young adult		8.27	1.82	0.001	3.99	−0.59	−12.169
	Adolescence	Young adult		3.44	1.82	0.134	−0.78	7.67	−6.608

The results of the post-hoc test revealed that, except for the MC of the low and moderate SES groups in young adulthood (*p* = 0.207), and the MC of adolescents and young adults (*p* = 0.134), all other groups showed significant differences in MC (*p* < 0.05). These differences were observed between low and high SES, moderate and low SES, and moderate and high SES in children and adolescents, high and low SES, and high and moderate SES in young adulthood, children and adolescents, and children and adults.

**Figure 3:**
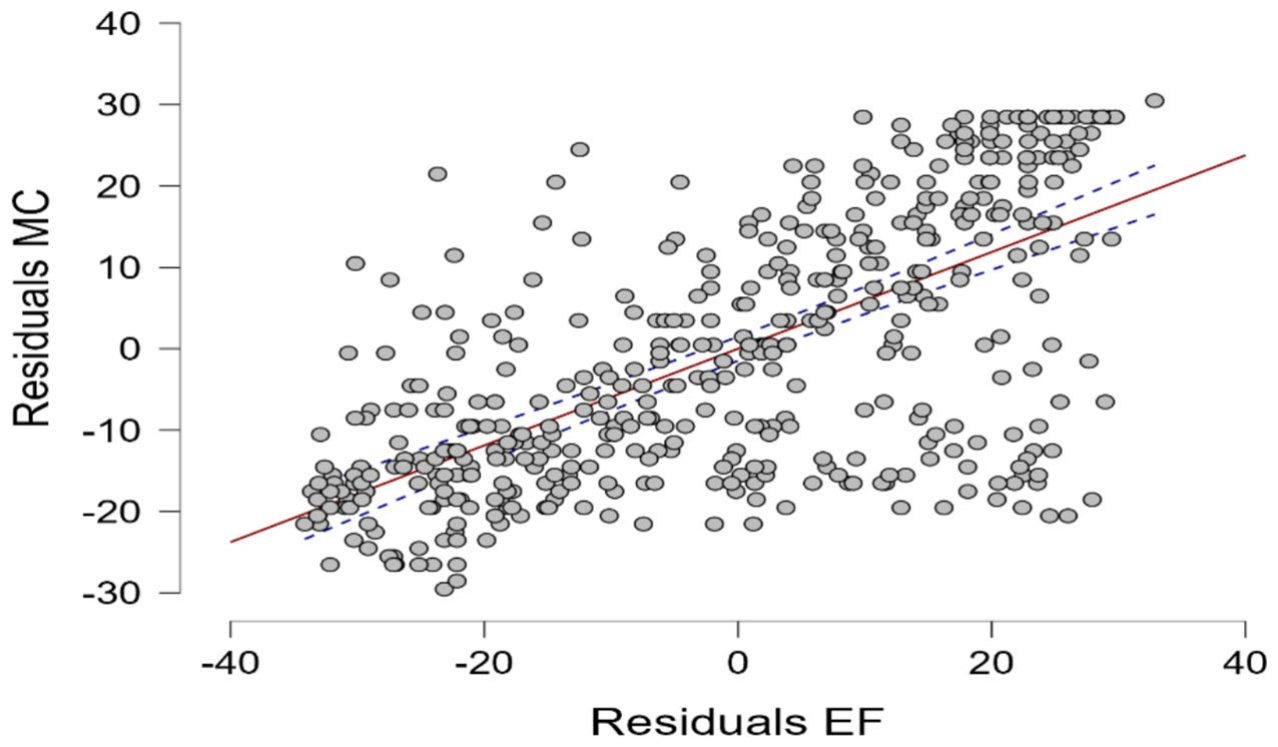
Association between MC and EF.

The correlation analysis revealed a significant relationship between MC and EF (*p* < 0.001, *r* = 0.671) (see Figure 3). The correlation results between MC and EF indicate a positive and significant relationship across all subgroups (*p* < 0.001). However, the strength of this correlation varies among different groups. In terms of gender, girls (*r* = 0.747) showed a stronger correlation compared to boys (*r* = 0.599). Regarding age, children (*r* = 0.715) and adolescents (*r* = 0.663) exhibited a stronger correlation than young adults (*r* = 0.650). For socioeconomic status (SES), the high SES group (*r* = 0.774) demonstrated a significantly stronger correlation compared to the moderate (*r* = 0.341) and low SES groups (*r* = 0.588).

Table 3 presents the results of the linear regression analyses for the study variables. The univariate regression analysis revealed a statistically significant association between MC and EF. Furthermore, in the multiple regression analysis, EF remained a significant predictor of MC participation after controlling for potential confounding variables, including age group, sex, and SES (Model 1). Based on Table 4, the strong positive effects of EF and high SES are particularly noteworthy, while age and sex group’s differences highlight the disadvantage of older groups relative to children, and girls to boys. These findings provide robust evidence for the influence of both individual and contextual factors on the dependent variable. To ensure the validity of the results, the assumptions of linear regression, including normality of residuals (assessed using Q-Q plots and the Shapiro–Wilk test), homoscedasticity (evaluated using residual scatter plots and the Breusch-Pagan test), absence of multicollinearity (confirmed by calculating VIF), and independence of errors (tested using the Durbin-Watson test), were examined and met. These checks confirmed that all assumptions were satisfied, and the results of the analyses are valid and reliable.

**Table 3:** Association of participants MC and EF in participants according to multiple regression analyses.

Dependent variable: MC	Univariate model *R*^2^ 0.451 Adjusted *R*^2^ 0.449		Model 1 b *R*^2^ 0.620 Adjusted *R*^2^ 0.615	
	*R*	*p* value	Standardized *β*	*p* value
EF	0.671	<0.001	0.444	<0.001

MC: MC; EF: EF; *R*^2^, Coefficient of determination; *β*, Regression coefficient.

Mode l was adjusted for Model 1 + AGE, SEX, and SES.

**Table 4:** Coefficients of variables.

Model 1	Unstandardized	Standard error	Standardized	*t*	*p*
EF	0.393	0.030	0.444	13.203	< 0.001
SES (average)	3.035	1.136		2.670	0.008
SES (high)	17.242	1.429		12.061	< 0.001
Age (adolescent’s)	−5.788	1.147		−5.047	< 0.001
Age (adults)	−7.205	1.158		−6.220	< 0.001
Sex (male)	2.172	0.938		2.315	0.021

## 4 Discussion

The primary objective of this study was to examine the effects of age, sex, and socio-economic status (SES) on motor competence (MC). The results indicated that sex did not have a statistically significant effect on MC. Although boys demonstrated higher MC compared to girls, this difference was not statistically significant. The main goal of the present study was to investigate the relationship between MC and executive function (EF). The results showed that the correlation between MC and EF was positive and strong (44%). Furthermore, we aimed to determine whether the relationship between MC and EF is influenced by age, sex, and SES. In this regard, the findings of the present study revealed both positive and negative effects of these factors on the correlation between EF and MC. However, it is important to emphasize that this study is correlational in nature, meaning that the observed relationship between MC and EF does not imply causation. While the 44% correlation suggests a meaningful association, it does not establish whether improvements in EF lead to better MC or vice versa. Other underlying factors, such as physical activity levels or cognitive stimulation, may also contribute to this relationship. Each of these objectives, along with their statistical results and references to previous studies, is detailed in the following sections.

### 4.1 The impact of age, sex, and SES on MC

#### 4.1.1 The effect of age on MC

Motor development models generally suggest that MC improves with age. However, this study, consistent with previous research (Jiménez-Díaz et al., 2015; Mohammadi Orangi et al., 2017, 2018, 2023a,b; Möller et al., 2021), demonstrates that children exhibit higher MC compared to adolescents and young adults. According to the analyses by Jiménez-Díaz et al. (2015) and Mohammadi Orangi et al. (2023a), one possible explanation for these findings is that children likely have more opportunities to develop motor skills than adolescents and young adults. Another potential explanation is that, after childhood, both structured and unstructured physical activities decrease or cease in individuals, making it challenging to maintain their level of MC. According to Newell (1972), and the ecological dynamics perspective (Button et al., 2020), individual constraints such as developmental changes and environmental constraints like reduced physical activity opportunities at older ages may account for these results.

Children benefit from increased interactions with their environment because they have sufficient time and space, such as at school or in playgrounds, to engage in play. This interaction leads to improved motor skills, as enriched environments for children provide time and space that serve as stimuli for movement (Jiménez-Díaz et al., 2015; Mohammadi Orangi et al., 2017, 2018, 2023a,b; Möller et al., 2021). Adolescents, while often burdened with academic responsibilities, still benefit from school environments and opportunities for play. Therefore, at this age, restrictive constraints like academic pressures or similar factors might result in lower MC compared to children (Jiménez-Díaz et al., 2015; Mohammadi Orangi et al., 2017, 2018, 2023a,b; Möller et al., 2021). However, adolescents still have access to school facilities and sports clubs, allowing them sufficient time to utilize these opportunities. These factors act as facilitating constraints, likely leading to better MC in adolescents compared to young adults. In older age groups, physical inactivity becomes a prevalent phenomenon, and motor activities decrease significantly (Jiménez-Díaz et al., 2015; Mohammadi Orangi et al., 2017, 2018, 2023a,b; Möller et al., 2021). This trend likely explains the lower MC observed in older individuals.

#### 4.1.2 The effect of SEX on MC

The results showed that sex is not a significant factor affecting MC. In a study conducted by Mohammadi Orangi et al. (2023a,b) in Iran, differences in MC between boys and girls were evident. However, in this study, these differences were not statistically significant among Turkish participants. This discrepancy in findings may be related to cultural influences and the opportunities available for physical activity in different countries. These differences may be due to cultural and social variations between Iran and Turkey. For instance, differing access to sports facilities and physical activity opportunities for girls in these two countries could influence the results. In Iran, particularly in areas with social restrictions for women, access to sports activities is limited, while in Turkey, girls may have more opportunities for participation.

Additionally, social trends and lifestyle changes in recent decades may have contributed to a reduction in gender differences in motor competence. With increased opportunities for girls to participate in physical activities and sports, especially in educational and recreational settings, the traditional gender gap in motor skill development may be narrowing. Furthermore, recent studies (Fathirezaie et al., 2022) suggest that environmental and upbringing factors play a crucial role in shaping motor competence. When parents and educators encourage both boys and girls equally to engage in motor activities, historical differences in motor skills may diminish over time. Moreover, it is important to note that the BOT-2 test assesses both fine and gross motor skills, which may contribute to the reduced or non-significant gender differences observed in this study. While studies specifically focusing on gross motor skills often report advantages for boys (Arifiyanti, 2020), the use of a comprehensive assessment tool like the BOT-2 may make these differences less pronounced.

According to Meier et al. (2021), women in Iran still lag behind men in terms of equal access to physical activity opportunities. Nevertheless, in the present study, MC in girls was lower than that of boys, even though the difference was not statistically significant. These findings indicate that in Turkey, efforts to equalize physical activity opportunities between men and women still require attention. The lower MC observed in girls, as measured by the standardized Bruininks-Oseretsky Test, even descriptively and without considering statistical differences, highlights that women continue to exhibit lower MC. This issue should be addressed as a social phenomenon and warrants further investigation.

#### 4.1.3 The effect of SES on MC

The results of this study also demonstrate that SES affects MC. Individuals with higher SES exhibit greater MC. These findings align with those of Möller et al. (2021) and a review of 59 studies (Barnett et al., 2016), which observed lower health-related behaviors, health awareness, and health outcomes among children from disadvantaged social backgrounds (Poulain et al., 2019).

Parents with lower SES levels may have less time, fewer financial resources, and lower motivation to promote active leisure activities and motor skills in their children (e.g., through frequent outdoor activities or participation in sports clubs). Specifically, individuals with higher SES typically have access to better sports facilities, professional coaches, and more suitable training programs. This access can enhance opportunities for learning and practicing motor skills, leading to improved MC (Barnett et al., 2016). Proper nutrition, which depends on a family’s financial capability, also plays a crucial role in physical growth and motor performance. Nutritional deficiencies can lead to reduced muscle development, endurance, and motor ability (Barnett et al., 2016). Families with lower SES may lack the time or resources to support their children in extracurricular activities (Poulain et al., 2019). Additionally, children from disadvantaged families may grow up in environments that lack sufficient space or opportunities for play and physical activity (Poulain et al., 2019). This can impact children’s confidence, motivation, and sense of self-efficacy, which, according to Stodden’s model, can influence MC (Stodden et al., 2008). Higher SES environments often provide more opportunities for informal motor skill learning, such as access to parks, sports clubs, or athletic groups. On the other hand, children in low-income environments may face social challenges, such as unsafe living conditions, which can limit their opportunities for physical activity (Möller et al., 2021). These factors underline the importance of addressing SES disparities to foster MC in children from all backgrounds.

### 4.2 The relationship between MC and EF

The results of this study revealed a strong and positive relationship between MC and EF, consistent with previous research. For instance, Albuquerque et al. (2022), Fathirezaie et al. (2022), reported a 38% correlation between EF and MC. In the current study, the correlation between MC and EF was reported as 44%, without considering confounding factors. It is important to note that the participants in Albuquerque’s study were aged 6 to 11, while the current study included a wider age range. This difference in participant age groups may explain the variation in reported correlation percentages, as diverse age ranges encompass different cognitive, psychological, and motor characteristics. Additionally, a meta-analysis by Gandotra et al. (2022), also emphasized a strong relationship between MC and EF. Similarly, in the study by Albuquerque et al. (2022) and Fathirezaie et al. (2022), a positive correlation between MC and EF was found among rural children in Iran. These studies support the findings of the present research, and it is similar to that. In interpreting these results, it can be argued that, according to the ecological dynamics perspective (Button et al., 2020), the development and competence of motor skills are influenced by multiple factors, one of which may be EF. This perspective highlights the interconnectedness of motor and cognitive domains in shaping human development.

EF encompasses high-level cognitive processes that play a crucial role in regulating and guiding complex activities (Gandotra et al., 2022). Similarly, MC requires precise coordination between cognitive and motor systems (Mohammadi Orangi et al., 2023a,b). The relationship between these two domains may stem from the demands of complex motor activities, which rely on abilities such as anticipation, decision-making, and rapid adaptation to environmental changes—all of which are dependent on EF (Gandotra et al., 2022).

This relationship can also be explained through the lens of the ecological dynamics perspective (Button et al., 2020), which posits that motor and cognitive development mutually influence each other. Within this framework, the environment plays a pivotal role, as environmental experiences can simultaneously enhance cognitive and motor capacities. For instance, engaging in challenging physical activities, such as team sports or complex motor games, not only demands control of bodily movements but also requires planning, coordination, and collaboration with others. This interaction between cognitive and motor systems helps establish stable and adaptive performance patterns, emphasizing the importance of integrating these systems in skill development. This interdependence highlights the need for holistic approaches in fostering both motor and cognitive growth.

The relationship between motor competence (MC) and executive function (EF) can be further explained from a neurocognitive and developmental perspective. Both MC and EF are associated with the development of brain regions that govern coordination and cognitive control. The cerebellum, which plays a key role in motor coordination, also contributes to higher-order cognitive functions like working memory and decision-making (Diamond, 2013). This dual role of brain structures highlights the interdependence between motor and cognitive systems, particularly in tasks that require complex motor planning, coordination, and cognitive control.

From a developmental perspective, both MC and EF follow a developmental trajectory that is influenced by age, experience, and environmental opportunities. As children engage in motor activities, their brain circuits involved in motor control and cognitive functions, such as the prefrontal cortex, become increasingly integrated. These interactions promote both motor skill development and the efficiency of executive processes such as attention, inhibition, and cognitive flexibility (Stodden et al., 2008). The interplay between these systems suggests that improvements in one domain may lead to enhanced performance in the other, especially during critical developmental periods.

### 4.3 The influence of age, sex, and SES on the relationship between MC and EF

The results of this study demonstrate that age, sex, and SES significantly influence the relationship between MC and EF. Specifically, sex has contrasting effects: males show a positive influence, whereas females have a negative impact. Although no significant differences in MC between boys and girls were observed, regression analysis highlighted the effect of sex on the relationship between MC and EF. Furthermore, age groups show varying impacts: children positively influence this relationship, while adolescents and young adults have a negative effect. Additionally, high SES contributes positively, whereas low SES has a negative influence.

These findings further emphasize Newell’s model and the ecological dynamics perspective (Button et al., 2020; Newell, 1972), underlining the importance of interactions between individual, environmental, and task constraints in shaping motor and cognitive development. EF was identified as a predictor of MC, explaining 44% of MC in the participants of this study. However, sex, age, and SES collectively account for 18% of the variance, leaving approximately 38% unexplained by these factors. This unexplained variance suggests the presence of other factors that warrant further investigation, potentially encompassing diverse individual, environmental, and task constraints.

Age, as a significant individual constraint, plays a crucial role in cognitive and motor abilities. At younger ages, the rapid development and greater flexibility of cognitive and motor systems can strengthen the relationship between these domains. However, at older ages, cognitive changes and physical limitations may weaken this relationship. These insights highlight the need for a holistic approach to understanding the interplay of motor and cognitive systems and their development across different demographic and environmental contexts (Mohammadi Orangi et al., 2023a,b).

Sex, within the ecological dynamics framework (Button et al., 2020), interacts dynamically with social and cultural institutions. In many societies, boys and girls experience different opportunities for physical activities and sports, which indirectly shape the development of motor skills and EF. These disparities highlight how sex influences the relationship between MC and EF through culturally and socially mediated access to environmental interactions. By shaping the individual’s engagement with physical activities and cognitive challenges, sex emerges as a key factor affecting this interplay.

SES serves as a critical environmental constraint, significantly impacting access to resources, educational opportunities, and physical activity participation. Individuals from higher SES backgrounds generally enjoy better access to facilities, professional coaching, and structured physical activities, which promote the development of MC and EF. Conversely, individuals from lower SES backgrounds face challenges such as limited access to sports and educational opportunities, negatively affecting the growth of these skills (Barnett et al., 2016).

These variations in resources and opportunities, framed within the dynamic interplay of individual and environmental constraints, significantly influence the relationship between MC and EF. The ecological dynamics perspective (Button et al., 2020; Newell, 1972) underscores how these constraints operate synergistically, shaping developmental pathways and determining the extent to which cognitive and motor skills can co-evolve across different socio-cultural contexts.

### 4.4 Strengths and limitations of the study

A key strength of this study was its examination of the impact of age, sex, and SES on MC across a wide range of ages and sex. Additionally, the study uniquely explored the relationship between MC and EF, as well as the influence of individual (age and sex) and environmental (SES) constraints on this relationship. The main limitation of this study was the assessment of overall scores for MC and EF. One reason for using overall scores was that, although the Bruininks-Oseretsky Test evaluates both fine and gross motor skills, the standardized score of this test is only available as a composite score. Since very few developmental tests consider a wide age range (4–21 years), using the total score of this test remains valuable. However, future studies are encouraged to examine the components of EF and their relationship with MC (including fine and gross motor skills, if possible). Another limitation of this study was that participants were selected from a city with limited ethnic and racial diversity. While university students (young adults) represented a mix of ethnicities, races, and cities, younger participants were predominantly local residents of İzmir. Thus, caution should be exercised when generalizing the findings to a larger population. Additionally, potential biases such as self-selection bias in participant recruitment or social desirability bias in SES reporting should be considered. These biases may have influenced the results and should be accounted for in future studies to ensure a more accurate representation of the sample.

Additionally, one limitation not fully addressed in the study concerns the socio-economic status (SES) measurement. The SES measurement relied on a limited number of indicators, primarily parental education, employment status, and household income. However, this approach did not take into account other potential SES indicators, such as living conditions or access to extracurricular activities, which may also influence motor competence and executive function. Although these SES factors were chosen based on prior research and established reliability, future studies could benefit from including additional SES measures to provide a more comprehensive view of socio-economic influences on MC and EF. Furthermore, cultural differences in motor skill development and potential measurement biases in the Stroop test should also be considered as potential alternative explanations for the results. Cultural factors such as access to physical activity opportunities, societal gender roles, and regional variations in educational practices may all contribute to differences in MC across different populations. Additionally, while the Stroop test is a widely validated tool for assessing executive function, variations in cultural or linguistic contexts could introduce biases that may affect the test’s reliability. These factors should be considered in future research to provide a more comprehensive understanding of the relationship between MC and EF.

Moreover, this study had a cross-sectional design, which prevents causal inferences. Although associations between MC and EF were discussed, some interpretations may imply causality, which should be addressed with caution. Future longitudinal studies are needed to determine whether improvements in MC lead to better EF over time. Another major limitation was the absence of data on physical activity levels. Since physical activity directly affects MC, failing to control for this variable is a fundamental limitation. Future studies should incorporate objective measures of physical activity, such as accelerometers, to control for its influence on MC and EF.

### 4.5 Future research directions

To address these limitations, future studies should conduct longitudinal research to determine causal relationships between MC and EF, as cross-sectional designs do not allow for strong causal inferences. Additionally, future studies should incorporate objective measures of physical activity, such as accelerometers, to control for the confounding effects of physical activity levels on MC and EF. Expanding SES measurement beyond parental education, employment, and income—by including factors such as living conditions and extracurricular opportunities—would provide a more comprehensive understanding of socio-economic influences. Furthermore, cross-cultural studies are needed to examine whether the absence of gender differences in this study is a regional trend or a global phenomenon. Finally, increasing sample diversity by recruiting participants from different socio-economic and ethnic backgrounds will enhance the external validity of future findings.

## 5 Conclusion

The results of this study revealed the impact of age, sex, and socio-economic status (SES) on motor competence (MC), as well as the relationship between MC and executive function (EF). Given that higher SES is associated with better motor competence, public health policies should implement more structured physical activity programs for children from lower economic backgrounds to ensure equal opportunities for motor and cognitive skill development. Age and SES were identified as significant factors influencing MC, whereas sex showed no significant effect. Another key finding was the positive and significant relationship between MC and EF, which can be explained by the ecological dynamics model and the interplay of individual and environmental factors.

These findings have important practical implications for education and sports training, particularly in designing programs that promote both motor competence and executive function. Schools can maximize the interaction between executive function and motor competence by integrating motor and cognitive exercises into their curricula. This includes activities such as movement-based games that require quick decision-making, balance training that enhances mental focus, and team exercises that simultaneously engage cognitive and motor skills. Additionally, sports organizations can create more inclusive and equitable opportunities for physical activity, ensuring that individuals of all socio-economic backgrounds have access to programs that support both motor and cognitive development.

Since the decline in motor competence among adolescents and young adults is a growing concern, age-specific interventions should be developed to prevent this decline. These interventions could include adaptable and challenging training activities aimed at improving balance, coordination, and reaction time. Furthermore, the effects of age, sex, and SES on the relationship between MC and EF suggest that various factors influence the concurrent development of cognitive and motor skills. To extend these findings, future research could employ longitudinal studies to examine the long-term effects of motor competence on executive function. Additionally, experimental designs that establish causal relationships between MC and EF would provide a deeper understanding of the underlying mechanisms. Further studies should also explore how cultural and environmental factors shape this relationship across diverse populations to provide more targeted policy recommendations.

## Data Availability

The raw data supporting the conclusions of this article will be made available by the authors, without undue reservation.
